# Surgical treatment of gastric cancer: A single center’s 15-year experience

**DOI:** 10.1016/j.clinsp.2025.100828

**Published:** 2025-11-21

**Authors:** Marcus Fernando Kodama Pertille Ramos, Marina Alessandra Pereira, Andre Roncon Dias, Osmar Kenji Yagi, Bruna de Camargo Nigro, Carolina Ribeiro Victor, Juliana Depra Panichella, Poliana Fonseca Zampieri, João Vitor Antunes Gregorio, Fauze Maluf-Filho, Edia Filomena Di Tullio Lopes, Evandro Sobroza de Mello, Ulysses Ribeiro-Junior

**Affiliations:** Instituto do Câncer do Hospital das Clínicas da Faculdade de Medicina da Universidade de São Paulo, São Paulo, SP, Brazil

**Keywords:** Gastric cancer, Gastrectomy, Operative surgical procedures, Multimodal therapy, Survival analysis

## Abstract

**Background:**

The treatment of Gastric Cancer (GC) has evolved with advances in multimodal therapy and minimally invasive surgery. This study aimed to provide an overview of the current treatment and survival outcomes of GC at a public high-volume cancer center during 15 years.

**Methods:**

Patients with gastric adenocarcinoma who underwent any surgical procedure between January 2009 and December 2023 were retrospectively included. To investigate temporal differences in patient characteristics and therapeutic approaches, the case cohort was divided into two time periods: Period I (2009–2016) and II (2016–2023).

**Results:**

A total of 1406 patients were included: 741 (52.7 %) were treated in Period I, and 665 (47.3 %) in Period II. The proportion of curative-intent surgeries remained stable (65.2% vs. 65.1), but diagnostic procedures increased (7.3 % vs. 12.2). Overall, 804 (57.2 %) patients underwent potentially curative gastrectomy. In Period II, patients had advanced ASA scores, more advanced clinical staging, and more frequent D1 lymphadenectomy (all *p* < 0.05). The use of minimally invasive surgery and the number of resected lymph nodes increased (both *p* < 0.001). Multimodal treatment became more frequent, with increased use of preoperative chemotherapy (*p* = 0.03). There were no significant differences in postoperative complications or 30- and 90-day mortality rates between the two periods. In multivariable analysis, advanced age, ASA III/IV, total gastrectomy, D1 lymphadenectomy, diffuse/mixed histology, advanced pT stage, and lymph node metastasis were independent factors associated with worse survival (all *p* < 0.05).

**Conclusion:**

Over 15 years, treatment evolved toward increased use of minimally invasive and multimodal approaches, with no impact on short-term surgical outcomes.

## Highlights

• 1406 patients with gastric adenocarcinoma underwent surgical treatment in 15 years.
• Minimally invasive surgery use has increased significantly.
• Diffuse-type tumors became more frequent.
• More lymph nodes were resected despite higher D1 lymphadenectomy rates.
• Multimodal treatment increased, with greater use of preoperative chemotherapy.

## Introduction

Gastric Cancer (GC) remains a major global public health concern.^1^ In Brazil, it ranks as the fourth most common cancer in men and the sixth in women. Surgery has remained the cornerstone of curative-intent treatment. For decades, tumor resection with negative margins combined with lymphadenectomy constituted the only available approach with curative potential.^2^

Over the past three decades, however, substantial advances have transformed the therapeutic landscape of GC. Chemotherapy, initially reserved for palliative care, has become an integral component of adjuvant and perioperative regimens, with demonstrated survival benefits.^3^ For early-stage tumors with a low risk of lymph node involvement, endoscopic resection has emerged as a viable alternative.^4^ Concurrently, surgical techniques have evolved significantly through the adoption of minimally invasive approaches ‒ such as laparoscopy and robotic-assisted surgery, individualized lymphadenectomy, and the use of intraoperative fluorescence imaging.^5^^,^^6^ These innovations have collectively contributed to surgical morbidity and mortality reduction.

Currently, the treatment of GC is inherently multimodal, requiring the coordinated efforts of multiple specialties, including medical oncology, surgical oncology, endoscopy, and radiology.^7^^,^^8^ In this context, the development of specialized cancer centers has been crucial for the implementation and consolidation of advanced, evidence-based therapeutic strategies.^9^

The Cancer Institute of the State of São Paulo (ICESP), established in 2008, serves as the primary cancer treatment facility within the Hospital das Clinicas complex of the University of São Paulo Medical School. Since its inception, ICESP has progressively integrated novel therapeutic modalities into the management of gastric cancer, establishing itself as a national reference center within the Brazilian Unified Health System (SUS) and playing a key role in the state oncology care network.^10^

The studied group previously reported the initial outcomes from the institute’s first decade of operation.^11^ However, a longitudinal assessment of evolving treatment strategies and outcomes was not yet feasible at that time. Therefore, the present study aims to provide an overview of the current treatment and short- and long-term outcomes of patients with gastric adenocarcinoma who underwent surgical treatment at a high-volume cancer center, over a 15-year period. To investigate temporal differences in patient characteristics and therapeutic approaches, the case cohort was divided into two equal time periods.

## Methods

All patients who underwent any surgical procedure for the treatment of gastric cancer between January 2009 and December 2023 at the Cancer Institute of the Hospital das Clínicas, University of São Paulo Medical School (ICESP), were included. Data were prospectively collected and stored in an institutional clinical database. Patients were excluded if they had tumors not originating in the stomach, underwent gastric resections for other malignancies, or had surgical procedures performed for benign conditions (e.g., peptic ulcers or gastrostomy).

To investigate temporal differences in the 15 years of treatment, patients were divided into two equivalent periods, as follows: January 2009 to June 2016 (Period I); and July 2016 to December 2023 (Period II) ‒ each period with 7 and a half years.

The procedures were considered as palliative (palliative gastrectomy, jejunostomy, bypass, cytoreduction), diagnostic (diagnostic laparoscopy or staging laparoscopy, with biopsy and/or lavage), and potentially curative (endoscopic resection, subtotal or total gastrectomy). Firstly, all cases treated at the service were analyzed and, subsequently, only cases submitted to gastrectomy (subtotal and total) with curative intent were evaluated.

Clinical variables included sex, age, Body Mass Index (BMI), American Society of Anesthesiologists (ASA) classification, and the Charlson-Deyo Comorbidity Index (CCI),^12^ calculated excluding gastric cancer as comorbidity. Preoperative laboratory data included serum albumin, hemoglobin (g/dL), and Neutrophil-to-Lymphocyte Ratio (NLR).

Preoperative staging was performed using Computed Tomography (CT) of the chest, abdomen, and pelvis, as well as upper gastrointestinal endoscopy. Patients who had tumors classified as locally advanced GC (cT2-T4 and/or cN+) with resectable disease and without signs of distant or peritoneal disease (cM0) were candidates for preoperative (perioperative or neoadjuvant) Chemotherapy (CMT). The indication for adjuvant therapy included all patients with a pathological stage > IB. Chemotherapy consisted of platinum-based (oxaliplatin or cisplatin) regimens. Surgical indication and technique were determined according to the guidelines of the Japanese Gastric Cancer Association and the Brazilian Gastric Cancer Association.^2^^,^^13^

The extent of gastric resection (total or subtotal) was determined by tumor location to ensure negative proximal margins. Resections were classified as R0, R1, or R2, indicating no residual tumor, microscopic residual tumor, or macroscopic residual tumor, respectively. The extent of lymphadenectomy was recorded as D1 or D2, based on the nodal stations resected.

Surgical complications were graded according to the Clavien-Dindo classification,^14^ with major complications defined as grades III to V. Thirty-day and 90-day mortality rates were assessed. Pathological evaluation followed the guidelines of the College of American Pathologists, and TNM staging was based on the 8th edition of the UICC/AJCC TNM classification.

Postoperative follow-up was conducted quarterly during the first year and biannually thereafter. Imaging and laboratory tests for recurrence detection were ordered based on the presence of clinical symptoms. Patients who missed follow-up appointments for more than 12-months were considered lost to follow-up.

This study was approved by the institutional Research Ethics Committee (Protocol NP993/16) and registered on Plataforma Brasil (CAAE: 2915,516.2.0000.0065).

### Statistical analysis

Descriptive statistics included absolute and relative frequencies for categorical variables and mean (with Standard Deviation, SD) or median (with Interquartile Range, IQR) for continuous variables. Categorical variables were compared using the Chi-Square test, while continuous variables were analyzed using Student’s *t*-test or Mann-Whitney *U test*, as appropriate.

Overall survival was calculated from the time of surgery until death. Disease-free survival was considered from the time of surgery until recurrence or death. Survival was estimated using the Kaplan-Meier method, and survival curves were compared using the log-rank test. Univariable and multivariable Cox proportional hazards regression models were used to identify factors associated with survival. Variables with statistical significance in univariate analysis were included in the multivariate model to identify independent prognostic factors. Patients alive at last contact were censored. All statistical analyses were two-sided, and all p*-*values < 0.05 were considered statistically significant. Analyses were performed using SPSS software, version 20.0 (SPSS Inc., Chicago, IL, USA).

## Results

**Fig. 1:**
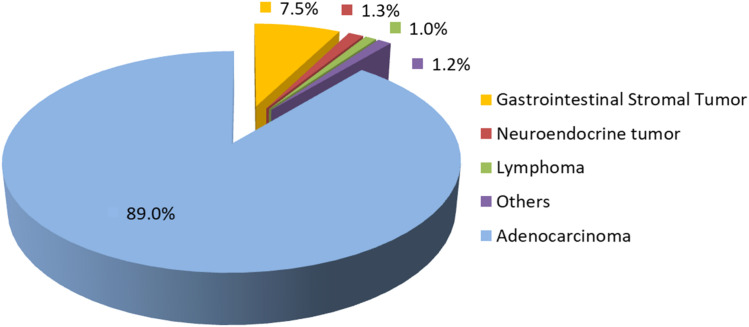
Frequency of gastric cancer cases according to histological type.

During the study period, 1580 patients with gastric cancer underwent surgical treatment. Among them, 1406 patients (89 %) had adenocarcinoma, 7.5 % Gastrointestinal Stromal Tumors (GIST), 1.3 % neuroendocrine tumors, 1 % lymphomas, and the remaining 1.2 % had other histological types (Fig. 1).

**Fig. 2:**
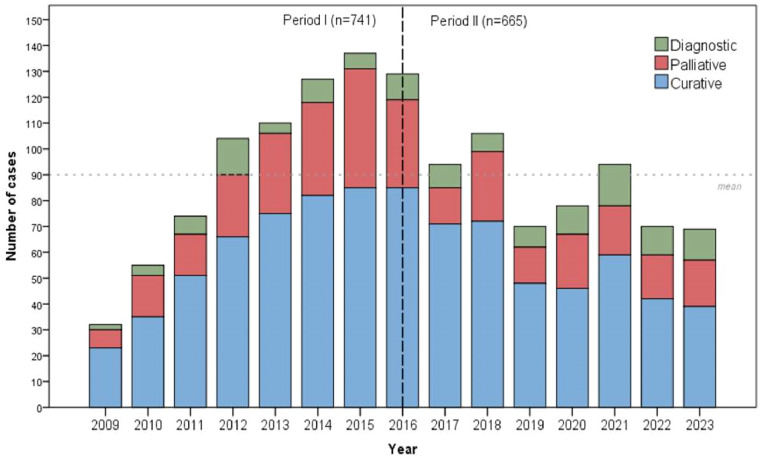
Frequency of gastric adenocarcinoma cases according to the type of procedure performed between 2009 and 2023, categorized as diagnostic, palliative, or curative.

Considering the 1406 patients with adenocarcinoma, 741 (52.7 %) patients were treated in Period I, and 665 (47.3 %) patients in Period II. Clinical characteristics of patients with adenocarcinoma are presented in Table 1. In the second period, the authors noted that a high number of younger patients were operated on, with no significant difference in sex distribution. A shift in the distribution of Lauren's histological subtypes was observed, with the intestinal type being more common in Period I and the diffuse type predominating in Period II. Additionally, G3 tumors were more frequently identified in Period II. There was a difference in the type of treatment (*p* = 0.004), with the proportion of curative-intent surgeries remaining stable between periods, while diagnostic procedures increased in Period II (Table 1 and Fig. 2). In addition, the use of minimally invasive surgery (laparoscopic and robotic) increased in Period II.

**Table 1:** Clinical characteristics and treatment of gastric adenocarcinoma patients according to the two treatment periods.

Variables	Period I	Period II	p
	*n* = 741 ( %)	*n* = 665 (%)	
Sex			0.319
Female	275 (37.1)	264 (39.7)	
Male	466 (62.9)	401 (60.3)	
Age (years)			0.043
Mean (SD)	63.4 (12.5)	62.1 (12.4)	
Lauren's Histological Type			<0.001
Intestinal	350 (57.5)	262 (45.6)	
Diffuse / Mixed	259 (42.5)	313 (54.4)	
Histological grade			<0.001
G1/G2	348 (56.9)	260 (45)	
G3	264 (43.1)	318 (55)	
cT			0.001
cT1	115 (15.5)	73 (11)	
cT2	175 (23.6)	125 (18.8)	
cT3	119 (16.1)	113 (17)	
cT4a	209 (28.2)	248 (37.3)	
cT4b	123 (16.6)	106 (15.9)	
cN			0.161
cN0	259 (35)	209 (31.4)	
cN+	482 (65)	456 (68.6)	
cM			0.591
cM0	549 (74.1)	501 (75.3)	
cM1	192 (25.9)	164 (24.7)	
cTNM			0.002
I	201 (27.1)	145 (21.8)	
II	136 (18.4)	103 (15.5)	
III	150 (20.2)	188 (23.3)	
IV	254 (34.3)	229 (34.4)	
Type of treatment			0.004
Curative	456 (65.2)	423 (65.1)	
Diagnostic	51 (7.3)	79 (12.2)	
Palliative	192 (27.5)	148 (22.8)	
Surgical access			<0.001
Open	610 (82.3)	404 (60.8)	
Endoscopic	42 (5.7)	16 (2.4)	
Robotic	14 (1.9)	53 (8)	
Laparoscopic	75 (10.1)	192 (28.9)

**Fig. 3:**
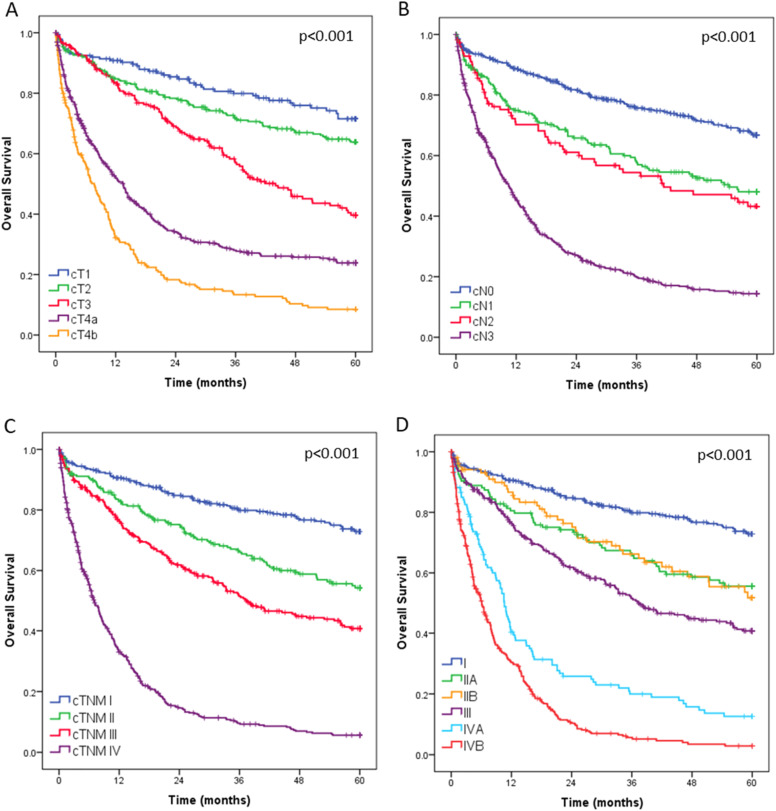
Overall survival of all 1408 patients with gastric adenocarcinoma according to clinical T (cT), clinical N (cN), and overall clinical stage (cTNM).

Overall survival curves for all 1408 operated patients, stratified by clinical T category (cT), clinical N category (cN), and final clinical stage, are presented in Fig. 3. During postoperative outpatient follow-up, 104 patients (7.4 %) were lost to follow-up before reaching 5-years.

### Curative surgical treatment

Of the 1406 patients with gastric adenocarcinoma, 804 (57.2 %) underwent potentially curative gastrectomy. Among these, 415 (51.6 %) were operated on during Period I and 389 (48.4 %) during Period II. In the second period, patients presented with higher ASA scores, more advanced cT and overall cTNM stages; there was an increased use of minimally invasive surgical approaches, and a higher frequency of D1 lymphadenectomy (Table 2).

**Table 2:** Clinical and surgical characteristics of patients undergoing curative-intent gastrectomy according to the two study periods.

Variables	Period I	Period II	p
	*n* = 415 (%)	*n* = 389 (%)	
Sex			0.401
Female	165 (39.8)	166 (42.7)	
Male	250 (60.2)	223 (57.3)	
Age (years)			0.066
Mean (SD)	63.1 912.7)	61.5 (12.2)	
Comorbidity (CCI)			0.500
CCI 0	274 (66)	248 (63.8)	
CCI ≥ 1	141 (34)	141 (36.2)	
BMI (kg/m²)			0.148
Mean (SD)	24.3 (4.8)	24.9 (5.0)	
Hemoglobin (g/dL)			0.914
Mean (SD)	12.2 (2.2)	12.2 (2.2)	
Albumin (g/dL)			0.396
Mean (SD)	4.0 (0.6)	3.9 (0.6)	
ASA			<0.001
I / II	336 (81)	227 (58.4)	
III / IV	79 (19)	162 (41.6)	
cT			0.002
cT1 / T2	217 (52.3)	160 (41.1)	
cT3 / T4	198 (47.7)	229 (58.9)	
cN			0.321
cN0	184 (44.3)	159 (40.9)	
cN+	231 (55.7)	230 (59.1)	
cTNM			0.001
I / II	263 (63.4)	200 (51.5)	
III / IV	152 (36.6)	189 (48.6)	
Type of resection			0.202
Subtotal	274 (66)	240 (61.7)	
Total	141 (34)	149 (38.3)	
Surgical access			<0.001
Open	367 (88.4)	249 (64)	
MI	48 (11.6)	140 (36)	
Lymphadenectomy			0.031
D1	65 (15.7)	84 (21.6)	
D2	350 (84.3)	305 (78.4)	

SD, Standard Deviation; ASA, American Society of Anesthesiologists.

Regarding pathological characteristics, there was an increase in the incidence of diffuse-type tumors, although the intestinal type remained the most common. Additionally, there was an increase in the number of resected lymph nodes (Table 3). The incidence of major postoperative complications did not differ significantly between the two periods (16.7 % vs. 15.9 %; *p* = 0.757). Thirty-day mortality was lower in the second period (2.6 % vs. 4.3 %), although the difference was not statistically significant (*p* = 0.181). The use of multimodal treatment increased in Period II, with a higher adoption of preoperative therapy and a decrease in the use of postoperative therapy.

**Table 3:** Pathological characteristics and postoperative outcomes of patients undergoing curative-intent gastrectomy according to the two periods.

Variables	Period I	Period II	p
	*n* = 415 ( %)	*n* = 389 (%)	
Lauren's Histological Type			0.032
Intestinal	239 (58)	189 (50.4)	
Diffuse / Mixed	173 (42)	186 (49.6)	
Tumor Size			0.543
Mean (SD)	4.8 (2.7)	4.7 (2.5)	
pT			0.664
pT1 / T2	174 (41.9)	169 (43.4)	
pT3 / T4	241 (58.1)	220 (56.6)	
pN			0.553
pN0	190 (45.8)	170 (43.7)	
pN+	225 (54.2)	219 (56.3)	
Number of Lymph nodes			<0.001
Mean (SD)	39.1 (17.8)	46.6 (19.8)	
pTNM			0.313
I / II	234 (56.6)	233 (59.9)	
III / IV	181 (43.6)	156 (40.1)	
Postoperative complication			0.757
None /Clavien I‒II	349 (84.1)	324 (83.3)	
Clavien III‒V	66 (15.9)	65 (16.7)	
Length of Hospital Stay (days)			0.334
Median (IQR)	9 (6 ‒ 13)	9 (6 ‒ 14)	
Mortality			
30-Day	18 (4.3)	10 (2.6)	0.181
90-Day	33 (8)	24 (6.4)	0.316
Preoperative chemotherapy^a^			<0.001
No	302 (84.6)	226 (67.7)	
Yes	55 (15.4)	108 (32.3)	
Postoperative chemotherapy^a^			0.025
No	127 (40.6)	153 (49.5)	
Yes	186 (59.4)	156 (50.5)	
Multimodal treatment^a^			0.033
No	153 (42.4)	120 (34.6)	
Yes	208 (57.6)	227 (65.4)	

SD, Standard Deviation.

a Only cases with indication to received CMT.

In a median follow-up time of 38.4 months (IQR 14 – 60), 298 patients died and 199 relapsed. The median follow-up for living patients was 60-months. The estimated 5-years OS and DFS for all curative resected patients were 57.3 % and 53.9 %, respectively. The estimated 5-year DFS rates of patients with pTNM stages I, II, and III were 73.9 %, 65.7 %, and 32.7 %, respectively.

**Fig. 4:**
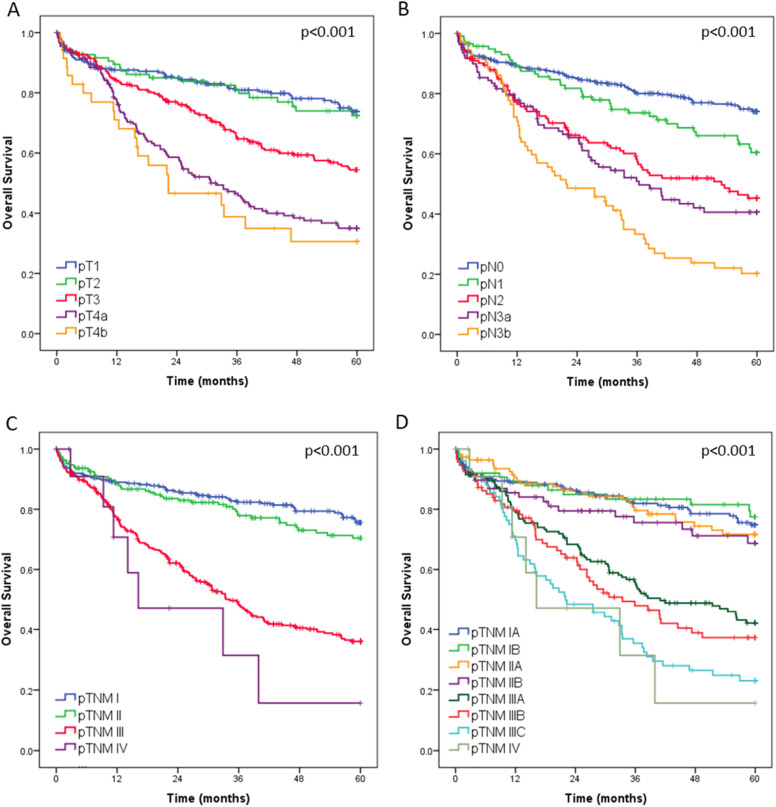
Overall survival for all curative patients with gastric adenocarcinoma according to the pT, pN, pTNM.

Regarding the OS, curves according to the pT, pN and pTNM categories are shown in Fig. 4. The estimated 5-year OS rates of patients with pTNM stages I, II, II and IV were 75.6 %, 70.4 %, 36.0 % and 1.6 %, respectively. The median OS was 34.2-months for Ptnm III and 16.2 for pTNM IV. Among patients with an indication for multimodal treatment (*n* = 708), those who received multimodal therapy had better survival compared to patients treated with surgery alone (*p* = 0.023).

In multivariable analysis, advanced age, ASA III/IV, total gastrectomy, D1 lymphadenectomy, diffuse/mixed histology, more advanced pT stage, and presence of lymph node metastasis were associated with worse overall survival (Table 4).

**Table 4:** Univariable and multivariable analysis for factors associated with overall survival – patients undergoing gastrectomy with curative intent.

Overall survival	Univariable			Multivariable		
Variables	HR	95 % CI	p	HR	95 % CI	p
Male (vs. female)	1.28	1.01 ‒ 1.62	0.037	1.29	0.99 ‒ 1.69	0.055
Age ≥ 65 (vs. < 65 years)	1.33	1.06 ‒ 1.67	0.013	1.36	1.05 ‒ 1.76	0.021
ASA III/IV (vs. ASA I/II)	1.91	1.51 ‒ 2.43	<0.001	1.40	1.03 ‒ 1.91	0.031
CCI ≥ 1 (vs. CCI 0)	1.37	1.09 ‒ 1.73	0.007	1.17	0.87 ‒ 1.56	0.305
Hemoglobin ≤ 11 g/dL (vs. > 11 g/dL)	1.61	1.27 ‒ 2.03	<0.001	1.13	0.85 ‒ 1.52	0.394
Albumin < 3.5 g/dL (vs. ≥ 3.5 g/dL)	1.91	1.46 ‒ 2.48	<0.001	1.25	0.92 ‒ 1.70	0.146
NLR ≥ 2.50 (vs. NLR < 2.50)	1.40	1.11 ‒ 1.75	0.004	1.11	0.86 ‒ 1.45	0.422
Total Gastrectomy (vs. subtotal)	1.71	1.37 ‒ 2.16	<0.001	1.60	1.24 ‒ 2.06	<0.001
D1 (vs. D2)	1.83	1.40 ‒ 2.38	<0.001	1.62	1.17 ‒ 2.24	0.004
Diffuse / Mixed (vs. others)	1.30	1.04 ‒ 1.63	0.023	1.46	1.13 ‒ 1.89	0.004
pT3/pT4 status (vs. pT1/pT2)	2.49	1.93 ‒ 3.23	<0.001	1.87	1.36 ‒ 2.59	<0.001
pN+ (vs. pN0)	2.65	2.05 ‒ 3.43	<0.001	1.92	1.41 ‒ 2.63	<0.001

CI, Confidence Interval; HR, Hazard Ratio.

## Discussion

The present study provides an overview of the treatment and survival outcomes during 15 years of GC treatment in Brazil from patients treated at a public referral center for oncological care, and updates the changes compared to a previous period.

The first notable finding was the relatively high frequency of non-adenocarcinoma histological subtypes, particularly Gastrointestinal Stromal Tumors (GISTs). While most large surgical series report proportions of adenocarcinomas above 95 %, in the present study, this rate was 89 %.^15^ The ICESP is a tertiary referral cancer center receiving patients from secondary hospitals throughout the state, which may contribute to this higher prevalence. Moreover, GIST diagnosis often requires Endoscopic Ultrasound (EUS), a resource still not widely available in the Brazilian public health system due to high costs, equipment fragility, and the need for specialized training.^16^ As a result, patients with suspected GIST are more frequently referred to high-complexity centers, leading to their overrepresentation in the cohort. Similarly, the care of patients with rare hereditary syndromes treated at other units within the Hospital das Clínicas, such as Multiple Endocrine Neoplasia type 1 (MEN-1) ‒ in which gastrin-producing pancreatic tumors may lead to secondary gastric neuroendocrine tumors ‒ may also explain this finding.

One apparent contradiction was the distribution of Lauren's histological subtypes. While diffuse-type tumors predominated in the overall adenocarcinoma cohort, intestinal-type tumors were more common among those resected with curative intent. This reflects a broader epidemiological shift seen in Brazil and globally.^17^^,^^18^ The intestinal subtype is typically linked to prolonged exposure to environmental carcinogens, including smoking, alcohol, processed foods, and *Helicobacter pylori* infection.^19^ The declining prevalence of these exposures is likely responsible for the global decrease in gastric cancer incidence, particularly for intestinal-type tumors. In contrast, diffuse-type tumors have maintained a relatively stable incidence and present a more aggressive biological behavior, which may explain their lower representation among patients eligible for potentially curative resection in this study.

The demographic characteristics of the adenocarcinoma population were consistent with the literature: the median age was 62 years, and there was a predominance of male patients. Although the mean age was statistically lower in the second period, this minimal difference is irrelevant in daily clinical practice. A more noteworthy observation was the worse clinical performance status, as assessed by the ASA score. This may reflect the impact of the COVID-19 pandemic. In a previous study, the studied group compared patients treated during and before the pandemic and observed the same trend.^20^ Patients who sought medical care during the pandemic were likely more symptomatic or debilitated and thus more willing to accept the risks of surgery. Furthermore, patients who delayed seeking medical care returned with a more compromised clinical status. Despite the difference in ASA classification, there was no significant change in the Charlson-Deyo comorbidity index between the periods, indicating a similar number of comorbidities but increased clinical impairment, either from more severe comorbidities or disease progression.

Clinical staging also worsened during the pandemic period, with a higher frequency of T3/T4 tumors, as previously described by the present group and corroborated by international studies. Therefore, the impact of the pandemic likely influenced the oncological characteristics observed in the second period, and this influence may persist in future analyses.^21^

The increased use of diagnostic laparoscopy in the second period is aligned with the more advanced clinical staging observed and the broader use of neoadjuvant chemotherapy.^22^^,^^23^ This procedure plays a critical role in detecting peritoneal dissemination, especially in tumors staged as > T3, and may alter treatment in up to 30 % of patients. In the studied institution, the implementation of a protocol for intraperitoneal chemotherapy has further increased the use of staging laparoscopy, as this is required for patient selection.^24^^,^^25^ Additionally, the increasing participation in international phase III neoadjuvant chemotherapy trials ‒ which include diagnostic laparoscopy as a prerequisite ‒ likely contributed to this trend.^26^^,^^27^

There was an increase in the use of minimally invasive surgery for patients undergoing resection with curative intent in the second period. Although laparoscopic gastrectomy has been described for over two decades, its broader adoption was limited due to concerns about oncological safety.^28^ Recent randomized controlled trials have demonstrated superior short-term outcomes and non-inferior oncological results for distal tumors, both early and advanced.^5^^,^^29^, ^30^, ^31^ For proximal lesions, evidence from randomized studies remains limited, though retrospective analyses have supported its safety and efficacy.^32^ With increasing surgical expertise and technological availability, the authors expect further expansion of minimally invasive approaches, although conventional surgery remains necessary in selected cases, including those involving large tumors, adjacent organ invasion, or extensive prior abdominal surgeries.

Another noteworthy observation was the increased number of D1 lymphadenectomy in the second period, despite the higher clinical stage. D2 lymphadenectomy is the standard approach in gastric cancer surgery, while D1 is reserved for selected early tumors not amenable to endoscopic resection.^33^ The increased frequency of D1 dissections appears contradictory but can be explained by the worse clinical status of patients in the second period. As previously described by the studied group, patients with compromised clinical performance may benefit from less extensive surgeries to reduce postoperative complications, even at the expense of long-term oncological outcomes.^34^

Despite the worst clinical performance in the second period, the authors observed a reduction in 30- and 90-day mortality, although not statistically significant. This may reflect institutional improvements in perioperative care and surgical experience.^35^^,^^36^ Another finding that supports the improvement in treatment quality is the increase in the number of dissected lymph nodes, despite the aforementioned rise in the frequency of D1 lymphadenectomies, which by definition involve the removal of fewer lymph nodes stations. This result may be attributed not only to greater surgical expertise but also to the active involvement of the pathology department.^37^

The survival analysis of the entire cohort clearly showed separation between clinical stages, supporting the accuracy of staging and appropriateness of therapeutic strategies applied. However, due to limited follow-up of patients in period II, it was not possible to compare long-term survival curves, as most patients have not yet reached the minimum follow-up period of three years. Unfortunately, the cause of death could not be reliably assessed, which precluded the analysis of cancer-specific survival.^38^

This study has some limitations inherent to its retrospective design, despite the prospective data collection. First, the specific cause of death could not be reliably determined, precluding the analysis of cancer-specific survival. Second, a loss to follow-up rate of 7.4 % was observed, which is considered moderate and acceptable for long-term observational studies. Nevertheless, it represents a relevant limitation that could affect the robustness of long-term survival estimates, despite appropriate censoring in survival analyses. Third, the present study included only patients who underwent surgical procedures. As a result, patients who received exclusively palliative treatment under the care of the medical oncology department were not evaluated. This exclusion may influence the baseline characteristics of the overall population; however, since most of these patients would be classified as clinical stage IV, the impact on survival analysis is likely minimal. Additionally, treatment decisions were not standardized by protocol and may have been influenced by institutional changes, multidisciplinary team experience, and evolving international guidelines throughout the study period. Unfortunately, a comparative survival analysis between Period I and Period II cannot be performed due to the significantly shorter follow-up in Period II. Any such analysis would risk introducing lead-time bias and underestimating long-term outcomes.

A strength of the present study is the inclusion of all patients who underwent surgical treatment for gastric cancer, regardless of intent. Most series report only those treated with curative intent, which underrepresents the full scope of surgical management.^39^^,^^40^ Additionally, all procedures were performed by the same surgical team, ensuring a high level of expertise and consistency in technique. Finally, all data were prospectively collected and maintained in a dedicated institutional database, ensuring data reliability and minimizing potential biases. This infrastructure has supported numerous graduate research projects over the past 15 years and continues to serve as a foundation for scientific research.

## Conclusion

Over the past 15 years, the treatment of gastric adenocarcinoma at the study center has evolved significantly. There was a greater use of minimally invasive surgery, increased adoption of multimodal therapy, and improved lymph node assessment. Despite more advanced cases in the second period, complication and mortality rates remained stable. Classical prognostic factors, for instance, ASA score, type of resection, lymphadenectomy, histology, and stage, continued to impact survival. These results support the importance of ongoing refinement in multidisciplinary gastric cancer care.

## CRediT authorship contribution statement

**Marcus Fernando Kodama Pertille Ramos:** Conceptualization, Methodology, Writing – original draft, Writing – review & editing. **Marina Alessandra Pereira:** Data curation, Formal analysis, Writing – review & editing. **Andre Roncon Dias:** Investigation, Visualization. **Osmar Kenji Yagi:** Investigation, Visualization. **Bruna de Camargo Nigro:** Investigation, Visualization. **Carolina Ribeiro Victor:** Investigation, Visualization. **Juliana Depra Panichella:** Investigation, Visualization. **Poliana Fonseca Zampieri:** Investigation, Visualization. **João Vitor Antunes Gregorio:** Investigation, Visualization. **Fauze Maluf-Filho:** Supervision. **Edia Filomena Di Tullio Lopes:** Supervision. **Evandro Sobroza de Mello:** Supervision. **Ulysses Ribeiro-Junior:** Supervision, Project administration.

## Declaration of competing interest

This article received no financial support, and the authors declare no conflict of interest.
